# Mentolabial angle and aesthetics: a quantitative investigation of idealized and normative values

**DOI:** 10.1186/s40902-017-0102-8

**Published:** 2017-02-05

**Authors:** Farhad B. Naini, Martyn T. Cobourne, Umberto Garagiola, Fraser McDonald, David Wertheim

**Affiliations:** 1 Consultant Orthodontist/Honorary Senior Lecturer, Kingston and St George’s Hospitals and St George’s Medical School, London, UK; 2 Professor and Head of Orthodontics and Craniofacial Development, King’s College London Dental Institute, London, UK; 3 Professor of Orthodontics, Department of Reconstructive and Diagnostic Surgical Sciences, University of Milan, Milan, Italy; 4 Professor of Orthodontics, King’s College London Dental Institute, London, UK; 5 Professor, Faculty of Science, Engineering and Computing, Kingston University, London, UK

**Keywords:** Mentolabial angle, Labiomental angle, Labiomental fold, Attractiveness, Facial, Profile aesthetics

## Abstract

**Background:**

This study is a quantitative evaluation of the influence of the mentolabial angle on perceived attractiveness and threshold values of desire for surgery.

**Methods:**

The mentolabial angle of an idealized silhouette male Caucasian profile image was altered incrementally between 84° and 162°. Images were rated on a Likert scale by pretreatment orthognathic patients (*n* = 75), lay people (*n* = 75) and clinicians (*n* = 35).

**Results:**

A mentolabial angle of approximately 107° to 118° was deemed the most attractive, with a range of up to 140° deemed acceptable. Angles above or below this range were perceived as unattractive, and anything outside the range of below 98° or above 162° was deemed very unattractive. A deep mentolabial angle (84°) or an almost flat angle (162°) was deemed the least attractive.

In terms of threshold values of desire for surgery, for all groups, a threshold value of ≥162° and ≤84° indicated a preference for surgery, although clinicians were least likely to suggest surgery. The clinician group was the most consistent, and for many of the images, there was some variation in agreement between clinicians and lay people as to whether surgery is required. There was even more variability in the assessments for the patient group.

**Conclusions:**

It is recommended that in orthognathic and genioplasty planning, the range of normal variability of the mentolabial angle, in terms of observer acceptance, is taken into account as well as threshold values of desire for surgery. The importance of using patients as observers in attractiveness research is stressed.

## Background

**Fig. 1:**
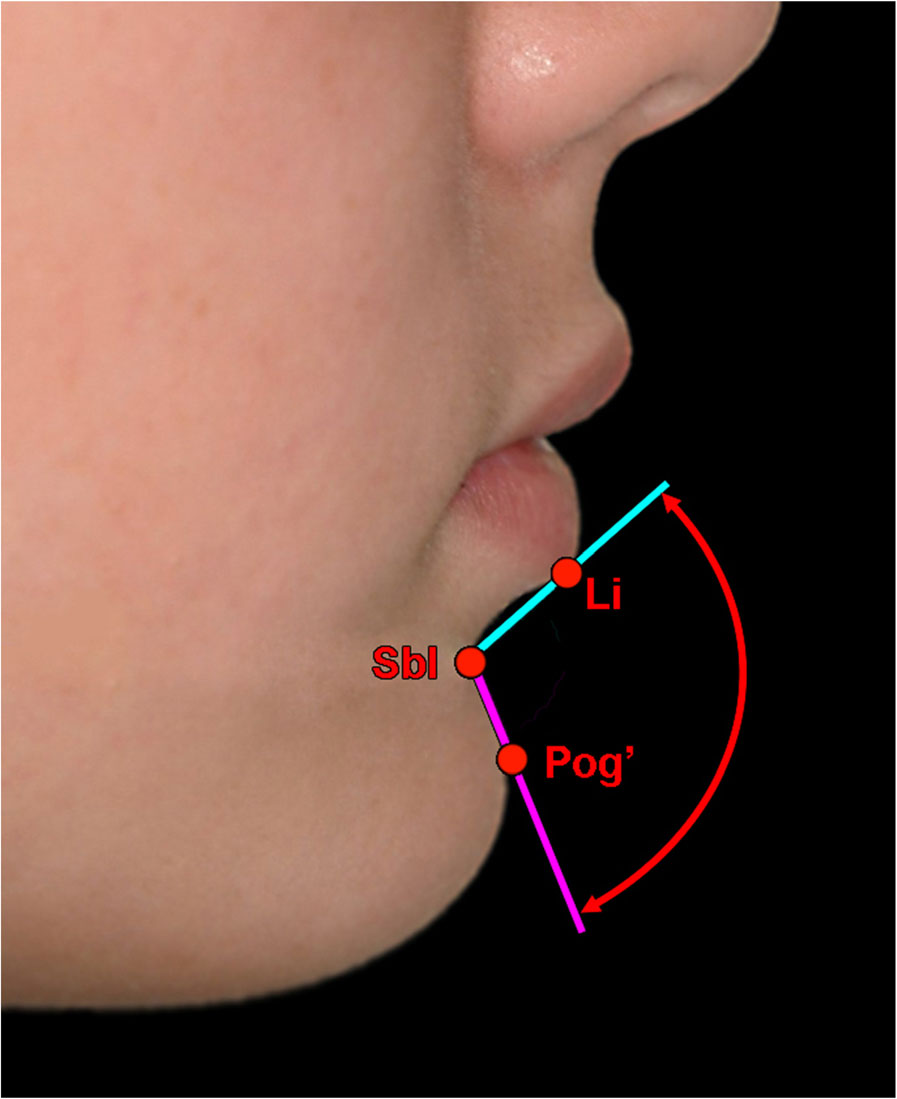
Mentolabial (labiomental) angle. This is the anterior angle formed by the intersection of a tangent to the lower lip (sublabiale to labrale inferius) and a tangent to the upper part of the soft tissue chin pad (sublabiale to soft tissue pogonion). *Li* labrale inferius, the midline point representing the mucocutaneous vermilion border of the lower lip; *Sbl* sublabiale, the midline point of greatest concavity on the facial contour of the lower lip between the labrale inferius and soft tissue menton. It is the deepest point of the mentolabial fold, also termed the soft tissue B point. *Pog’* soft tissue pogonion, the most prominent midline point of the soft tissue chin pad

The mentolabial (or labiomental) region is evident in frontal and profile views and forms the transition from the lower lip to the soft tissue chin. The morphology of this region is one of the most important aesthetic parameters of the facial profile, and an observer’s visual perception of the lower face is often drawn to this region [1]. The mentolabial angle, also termed the labiomental angle, is a potentially important factor in the perception of facial profile attractiveness. It is the anterior angle formed by the intersection of a tangent to the lower lip (sublabiale to labrale inferius) and a tangent to the upper part of the soft tissue chin pad (sublabiale to soft tissue pogonion) (Fig. 1) [2].

The mentolabial region and angle must be carefully evaluated when planning orthognathic surgery, particularly mandibular surgery, osseous genioplasty or the placement of chin implants. The upper component of the angle may be affected by mandibular incisor proclination during class III decompensation, which will, to some extent, lead to concomitant proclination of the lower lip and thereby reduction of the mentolabial angle. Any surgical procedure that increases mentolabial height will increase the mentolabial angle and thereby open the fold, e.g. mandibular advancement, to a three-point (tripod) landing, antero-inferior advancement genioplasty or clockwise rotation of the mandible or maxillomandibular complex. The opposite is also true; any procedure that reduces lower face height tends to deepen the mentolabial fold and decrease the angle. Therefore, the aesthetics of this region are vitally important both in terms of dentofacial surgical diagnosis and treatment planning [3].

The principal aim of this investigation was to evaluate quantitatively the influence of lower facial profile aesthetics as represented by the mentolabial angle on perceived attractiveness. The relationship between the mentolabial angle and attractiveness was recorded to ascertain the range of normal variability, in terms of observer acceptance, and to determine the clinically significant threshold value or cut-off point, beyond which the angle is perceived as unattractive and surgical correction is desired. The perceptions of patients, clinicians and lay people were compared for these different variables.

## Methods

Ethical approval was sought and granted for the study by the National Research Ethics Service; NRES (UK); REC reference: 06/Q0806/46.

Two-dimensional profile silhouettes are used routinely to assess the perceptions of facial attractiveness [4, 5]. A profile silhouette image was created with computer software (Adobe® Photoshop® CS2 software). The image was manipulated using the same software to construct an “ideal” profile image with proportions, and linear and angular soft tissue measurements [6–8], based on currently accepted criteria for an idealized Caucasian male profile, as previously described [5].

**Fig. 2:**
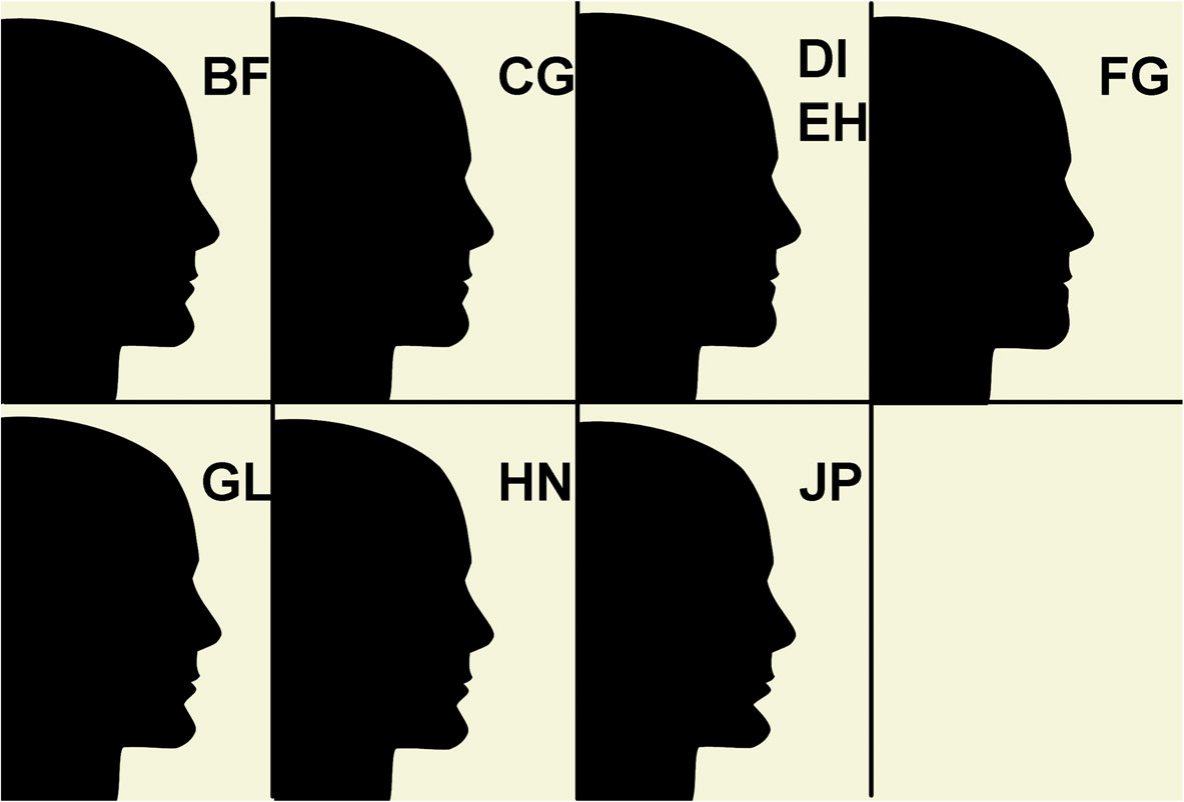
The mentolabial angle of the idealized profile image was altered incrementally, creating a series of images

The mentolabial angle of the idealized image (image BF 118°) was altered incrementally from 84° to 162°, in order to represent variations in the angle, ranging from excessive to an almost flat mentolabial morphology (Fig. 2).

Based on the results of a pilot study and power calculation, 185 observers took part in the study, separated into three groups (pretreatment orthognathic patients, lay people and clinicians; Table 1), with the following selection criteria:

**Table 1:** Observer demographics

Observer group	Number	Mean age (in years)	95% CI	Age range	Gender (% male)	Ethnicity (% White)
Orthognathic patients	75	22	20-24	13–60	42	66
Lay people	75	31	28-35	16–79	31	49
Clinicians	35	31	30-33	24–39	33	72

- Patients: pretreatment (only one consultation appointment), primary concern was facial appearance, no previous facial surgical treatment, no history of facial trauma and no severe psychological issues.
- Lay people: no previous facial surgery, deformities or history of facial trauma.
- Clinicians: involved in the management of patients with facial deformities and included 19 maxillofacial surgeons and 16 orthodontists, with 1–16 years of experience in the clinical management of patients requiring orthognathic and facial reconstructive surgery.

Each observer was given a questionnaire and asked to provide the following information: age, gender, ethnic origin (White or non-White), how would you rate the attractiveness of your facial appearance and how important do you think it is to have an attractive facial appearance. An instruction sheet accompanied the questionnaire, asking the observers to rate each image in terms of facial attractiveness using the following rating scale:

1. Extremely unattractive
2. Very unattractive
3. Slightly unattractive
4. Neither attractive nor unattractive
5. Slightly attractive
6. Very attractive
7. Extremely attractive

Observers were also asked whether they would consider surgery to correct the appearance if this was their facial appearance (yes or no).

**Fig. 3:**
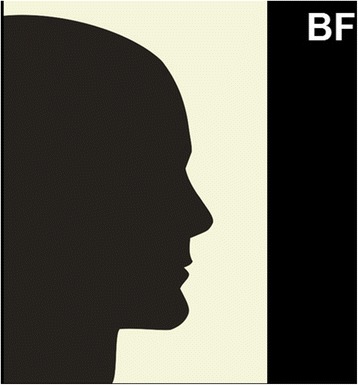
An example of an image viewed by study observers on the monitor during data collection

The images were placed in random order into the software application Microsoft PowerPoint® (Microsoft Corporation, USA). Each image was identified by a randomly assigned double letter in the top right corner of the screen (e.g. BF and CG; Fig. 3). A duplicate image assessed intra-examiner reliability (images DI and EH). Each observer sat undisturbed in the same room in front of the same computer and 17-in. flat screen monitor. The presentation and the images were created in such a way that each of the profile silhouette images, when viewed on the monitor, had the same dimensions as a normal human head, based on an average lower facial height, reducing the potential effect of image size on observer perception. Each observer examined the images in the PowerPoint® presentation by pressing the “Page Down” button on the keyboard, in their own time.

The Likert-type rating scale used is largely accepted in the psychology literature as the most useful rating method [9]. The 7-point Likert scale described above was used by each observer to rate each image in terms of attractiveness.

### Statistical analysis

The median and interquartile observer ratings were calculated for each angle and for each observer group; these descriptive statistics were calculated using software that we developed using MATLAB (The MathWorks Inc., Natick, MA, USA). Additionally, data were modelled by curve fitting performed using MATLAB. Similarly, the software calculated the proportions in each group suggesting a desire for surgery. Additional paired *t* tests were performed using Minitab version 16 (Minitab Inc., State College, PA, USA) following application of the Ryan-Joiner test in Minitab used to examine if data were consistent with a normal distribution.

## Results

### Reliability analysis

Table 2 shows the first and third quartile rankings of the Likert score. The results indicate that there was generally good agreement in the three observer groups. The median interquartile range for all three groups was 1 and the maximum interquartile range was 2.

**Table 2:** First and third quartile rankings of the Likert score

		First quartile			Third quartile		
Image	Angle (°)	Patients	Lay	Clinicians	Patient	Lay group	Clinician
BF	118	4	5	4.25	5	6	6
CG	127	4	4	4	5	6	5
DI	140	3	3	4	4	5	4.75
EH	140	3	4	3	4.75	5	5
FG	162	2	2	3	3	4	4
GL	107	4	4	4	5	6	5
HN	98	2	3	3	3.75	4	4
JP	84	2	2	2	3	4	3

### Perceived attractiveness of images

In Table 3, the median attractiveness rating of the observers on a Likert scale from 1 to 7 is shown, where 1 indicates “extremely unattractive” and 7 indicates “extremely attractive”. A mentolabial angle outside the range of 107° to 140° was associated with a reduction in the median attractiveness scores to below 4 in all three groups of observers except for the lay group at 98°. All groups have the same median attractiveness score for the identical images (DI and EH), thus again indicating good repeatability.

**Table 3:** Median attractiveness observer ratings on the Likert scale

		Median score		
Image	Angle (°)	Patients	Lay	Clinicians
BF	118	5	5	5
CG	127	4	5	5
DI	140	4	4	4
EH	140	4	4	4
FG	162	3	3	3
GL	107	5	6	5
HN	98	3	4	3
JP	84	2	3	3

### Most attractive and least attractive images

Table 4 demonstrates the data in rank order from most to least attractive, sorted on the basis of responses from the clinician group then lay group. Tables 5 and 6 demonstrate the proportion expressed as a percentage of each observer group suggesting that surgery is required. The results indicate that clinicians were generally least likely to suggest surgery for varying degrees of mentolabial angle except for the 84° category. Images DI and EH were identical, and so, repeatability of the 35 clinicians’ assessments was excellent with only one clinician suggesting surgery for one of the images. For the 75 lay people, the assessment of the two repeated images was also similar (17 and 19%), which is also seen in the group of 75 patients (both 29%). For many of the images, there was generally reasonable agreement amongst clinicians and lay people as to whether surgery is required. Taking 50% (i.e. majority) of each observer group as a cut-off where the majority suggested surgery, for all three groups, the only category for desire for surgery was at an angle of 84°.

**Table 4:** Data in rank order from most to least attractive (clinician ranking first)

		Median score		
Image	Angle (°)	Patients	Lay	Clinicians
GL	107	5	6	5
BF	118	5	5	5
CG	127	4	5	5
DI	140	4	4	4
EH	140	4	4	4
HN	98	3	4	3
FG	162	3	3	3
JP	84	2	3	3

**Table 5:** Proportion expressed as a percentage of each observer group suggesting a desire for surgery

		Suggesting surgery		
Image	Angle (°)	Patients	Lay	Clinicians
BF	118	15	9	0
CG	127	20	4	0
DI	140	29	17	3
EH	140	29	19	0
FG	162	60	60	40
GL	107	24	7	0
HN	98	59	43	23
JP	84	69	63	80

**Table 6:** Proportion expressed as a percentage of each observer group suggesting a desire for surgery in rank order

		Suggesting surgery		
Image	Angle (°)	Patients	Lay	Clinicians
CG	127	20	4	0
GL	107	24	7	0
BF	118	15	9	0
EH	140	29	19	0
DI	140	29	17	3
HN	98	59	43	23
FG	162	60	60	40
JP	84	69	63	80

For observers who considered attractiveness to be important (>2), Table 7 indicates the proportion suggesting surgery. For patients 68/75, for lay people 71/75, and all clinicians considered attractiveness to be important. Thus, a mentolabial angle of 84° was again the only category associated with all groups suggesting surgery.

**Table 7:** Proportion of observers suggesting surgery who considered attractiveness to be important

		Suggesting surgery		
Image	Angle (°)	Patients	Lay	Clinicians
BF	118	15	10	0
CG	127	21	4	0
DI	140	31	18	3
EH	140	31	20	0
FG	162	62	59	40
GL	107	26	3	0
HN	98	62	41	23
JP	84	71	62	80

For those who did not consider attractiveness to be important (seven patients and four lay people), Table 8 summarizes the proportion desiring surgery; the table has no column for clinicians as all considered attractiveness to be important. Clearly, the lay observer number is low in this category.

**Table 8:** Proportion of observers suggesting surgery who did not consider attractiveness to be important

	Suggesting surgery		
Image	Angle (°)	Patients	Lay
BF	118	14	0
CG	127	14	0
DI	140	14	0
EH	140	14	0
FG	162	43	75
GL	107	0	75
HN	98	29	75
JP	84	57	75

## Discussion

Planning orthognathic surgery requires the determination and validation of correct mentolabial morphological relationships, which requires two sources of information [10]. Age-, gender- and ethnicity-specific population averages based on anthropometric data allow comparison of a patient’s mentolabial measurements and proportions to the population norms. No longitudinal data is available for the mentolabial angle, but there is some cross-sectional data available [7]. Additionally, the perceived attractiveness of the proportions and morphological relationships should be confirmed by the judgement of patients and the lay public and ideally compared to the judgement of treating clinicians. This was the main purpose of this investigation.

The results of this investigation demonstrated that increasing the mentolabial angle deviation in either direction from an angle of 107° to 118° (images BF and GL, respectively) was associated with a reduction in the median attractiveness scores in all three groups of observers. The highest attractiveness scores were for image GL (107°), closely followed by image BF (118°) and image CG (127°). An angle of 140° (images DI and EH) was deemed to be neither attractive nor unattractive, i.e. essentially acceptable, even if not attractive. However, from a mentolabial angle of 84° and below, and 162° and above, the images were viewed as unattractive by all observer groups. It may be postulated that angles outside these ranges are likely to be perceived as unattractive by all groups, with greater deviations leading to potentially progressively reduced perceptions of attractiveness.

In terms of desire for surgical correction, the results indicate that clinicians were generally the least likely to suggest surgery for varying degrees of mentolabial angle. Although there was some general agreement in the three observer groups, particularly for the more extreme angles, there appears to be a higher degree of agreement amongst clinicians, and the reason for this may be the potentially higher critical capabilities of clinicians resulting from their training. This stresses the importance of using patients as observers in facial attractiveness research.

As with other facial parameters, it is generally acknowledged that the mentolabial angle has a range of normal individual variability. As a starting point, for comparative purposes and by way of contrast, it is useful to look at the mentolabial angle in idealized images from classical and Renaissance art and sculpture (Table 9). The first known treatise on ideal human proportions was written by the Greek sculptor Polykleitos of Argos. Unfortunately, no copies of this book exist. However, it is known, based on evidence from the physician Galen, that Polykleitos based his most important statue, the Doryphoros, on his treatise. The mentolabial angle in the Doryphoros statue is approximately 105°. The statue of Heracles has a more open angle of 125°, and Hermes at 109°, yet all these idealized male statues demonstrate mentolabial angles within the range found as most attractive in this investigation. The idealized female profile of Venus de Milo demonstrates a somewhat deeper mentolabial angle of 95°. From a number of idealized male and female profile images painted or sculpted in the Renaissance, the mentolabial angle is again within the range of 95 to 128°. A common denominator in the morphology of the mentolabial region in these images is that there is a relatively soft, S-shaped curve in the transition from the lower lip to the chin.

**Table 9:** The mentolabial angle in idealized images from classical and Renaissance art and sculpture

Artwork	Artist	Era	Mentolabial angle (°)
Doryphoros (Pompeii, now in Naples)	Polykleitos of Argos	Classical Greece	105
Heracles (Naples)	Polykleitos of Argos	Classical Greece	125
Hermes	Apollonius	Classical Greece	109
Aphrodite of Milos (Venus de Milo)	Alexandros of Antioch	Hellenistic Greece	95
Head of a youth in profile (male head)	Leonardo da Vinci	Italian Renaissance	95
Study of the valves and muscles of the heart (male head in profile)	Leonardo da Vinci	Italian Renaissance	105
Woman’s head in profile	Leonardo da Vinci	Italian Renaissance	128
La Bella Principessa	Leonardo da Vinci	Italian Renaissance	130
Idealized head of a woman	After Leonardo da Vinci (unknown artist)	Italian Renaissance	99
Head of a woman in profile	Giovanni Antonio Boltraffio	Italian Renaissance	118
David	Michelangelo Buonarroti	Italian Renaissance	100
Primavera (middle sister, profile)	Botticelli	Italian Renaissance	117
Woman’s profile (from The Three Ages of Man)	Titian	Italian Renaissance	116

Additionally, a number of modern surgical authorities have provided “ideal” values for the mentolabial region or angle, based on anecdotal evidence and the “good eye” of the respective surgeon. Interestingly, in their “aesthetic triangle”, Powell and Humphreys [11] have not included the mentolabial angle. However, they have suggested that the “ideal” depth of the mentolabial fold should be 4 mm from a vertical line drawn between the labrale inferius and soft tissue pogonion. Papel [12] corroborated the 4-mm depth value provided by Powell and Humphreys [11] but again did not discuss the mentolabial angle. Lehocky [13] provided the ideal values as 113 ± 21° in men and 121 ± 14° in women, based on anecdotal opinion. However, similar to Powell and Humphreys [11], he suggested the depth of the mentolabial fold to be 4–6 mm and that it should be deeper for men than women. Legan and Burstone [14] also suggested an ideal mentolabial fold depth of 4 ± 2 mm, based on analysis of 20 white Caucasian males and 20 females with class I occlusions and “facial proportions that were determined to be within normal limits”. Naini [2] provided a mentolabial angle range of 115°–145° for males and 120°–130° for females but stressed the importance of dividing the angle into upper and lower component parts by a true horizontal line drawn through the sublabiale. Nanda et al. [15] determined that at 18 years, the mean value of the mentolabial angle was 125.1° ± 12.9° in males and 127.1° ± 12.9° in females. Average values, based on anthropometric studies by Farkas [7], for adult North American Whites are 113.5 ± 20.7° in males and 121.4 ± 14.4° in females. However, Farkas [7] also provided male values of 147.2 ± 20.7°, albeit “indirectly derived”. Information in the literature regarding ethnic variability of the mentolabial angle is limited. Wen et al. [16] identified only one study for African individuals and three studies for Asian (Far Eastern) individuals. The African study, by Farkas et al. [17], based on measurement of 54 adult males and 123 adult females, provided a mentolabial angle of 130.2° (95% confidence interval of 122.0°–138.4°) for males and 129.0° (95% confidence interval of 120.1°–136.3°) for females. Wen et al. [16] identified only three studies on “Asian” (Far Eastern) populations, based on data from 185 adult males and 223 adult females, providing the values for a mentolabial angle of 134.8° (95% confidence interval of 128.8°–40.4°) for males and 133.4° (95% confidence interval of 128.3°–138.5°) for females. On the whole, the aesthetic analysis of the mentolabial region is under-discussed in the literature when compared with nasal aesthetic analysis.

It is important to bear in mind that the profile silhouette image created for this investigation was based on North American white adult male proportions and normative values. As such, it is not generalizable to different ethnic groups and the data may not be directly relevant to other ethnic groups, though it does provide an insight into how different ethnic groups view Caucasian faces. It would be interesting to repeat the study using images from different ethnic groups. It would also be useful to obtain data on the potential relationship between perceived attractiveness of the mentolabial angle and lower anterior face height.

## Conclusions

The results demonstrate that, based on the images used in this investigation, a mentolabial angle of approximately 107° to 118° was deemed the most attractive, with a range of up to 140° deemed acceptable. Angles above or below this range are perceived as unattractive, and anything outside the range of below 98° or above 162° is deemed very unattractive. A deep mentolabial angle (84°) or an almost flat angle (162°) was deemed the least attractive.

In terms of threshold values of desire for surgery, for all groups, a threshold value of ≥162° and ≤84° indicated a preference for surgery, although clinicians were least likely to suggest surgery. The clinician group were the most consistent, and for many of the images, there was some variation in agreement between clinicians and lay people as to whether surgery is required. There was even more variability in the assessments for the patient group. This stresses the importance of using patients as observers, as well as lay people and clinicians, in facial attractiveness research.
